# Retraction: Sodium nitroprusside application improves morphological and physiological attributes of soybean (*Glycine max *L.) under salinity stress

**DOI:** 10.1371/journal.pone.0272180

**Published:** 2022-08-03

**Authors:** The PLOS ONE Editors

The *PLOS ONE* Editors retract this article [1] because it was identified as one of a series of submissions for which we have concerns about authorship, competing interests, and peer review. We regret that the issues were not addressed prior to the article’s publication.

ZJ, NH, and SAHB did not agree with the retraction. HAF, FI, MNH, JL, SR, WH, HY, SM, AK, RNAQ, and MSA either did not respond directly or could not be reached.

## References

[1] Jabeen Z, Fayyaz HA, Irshad F, Hussain N, Hassan MN, Li J, et al. (2021) Sodium nitroprusside application improves morphological and physiological attributes of soybean (*Glycine max* L.) under salinity stress. PLoS ONE 16(4): e0248207. 10.1371/journal.pone.0248207 33861749 PMC8051766

